# Supplementary material for: Copolymers of 4-Trimethylsilyl Diphenyl Acetylene and 1-Trimethylsilyl-1-Propyne: Polymer Synthesis and Luminescent Property Adjustment
Source: Molecules. 2022 Dec 21;28(1):27. doi: 10.3390/molecules28010027 (PMC9822087; doi:10.3390/molecules28010027)
Supplement: Supplementary file 1 [file molecules-28-00027-s001.zip › molecules-2109221-supplementary.pdf]

Supporting Information for

Copolymer of 4-trimethylsilyl diphenyl acetylene and 1-trimethylsilyl-1-propyne: Polymer synthesis and luminescent property adjustment

Tanxiao Shen [1], Manyu Chen [1], Haoke Zhang [1,2,3], Jing Zhi Sun [1,2,*] and Ben Zhong Tang [1,4]

[1] MOE Key Laboratory of Macromolecular Synthesis and Functionalization, Department of Polymer Science and Engineering, Zhejiang University, Hangzhou 310027, China
[2] Centre of Healthcare Materials, Shaoxing Institute, Zhejiang University, Shaoxing, 312000, China
[3] ZJU-Hangzhou Global Scientific and Technological Innovation Center, Hangzhou 311215, China
[4] Shenzhen Institute of Aggregate Science and Technology, School of Science and Engineering, The Chinese University of Hong Kong, Shenzhen 518172, China
* Correspondence: sunjz@zju.edu.cn Tel.: 0086 13958091775.

Content

Figure S1. FTIR spectra of TMSP, PTMSP, TMSDPA and PTMSDPA S2

Figure S2. FTIR spectra of PTMSP, P**1**, P**2** and PTMSDPA S3

Figure S3. FTIR spectra of P**5**, P**6**, P**7**, P**8** and P**9**........ S4

Figure S4. ^{1}H NMR spectra of PTMSP, P**7** and PTMSDPA. S5

Figure S5. UV-vis spectra of P**10** and P**11** in THF solution S6

Figure S6. PL spectra of P**10** and P**11** powders.. .... S6

Figure S7. Fluorescence photos of P6 in THF solution (10^{-3} M) and powder under 365 nm light S6

Figure S8. PL behavior of P**7** and the plot of peak intensity of P**7** in THF/water mixtures with different water fractions........ S7

Figure S9. PL behavior of PTMSDPA and the plot of peak intensity of P**7** in THF/water mixtures with different water fractions........ S7

Table S1. The quantum yield for the suspensions of PTMSDPA, P**6** and P**7** S8

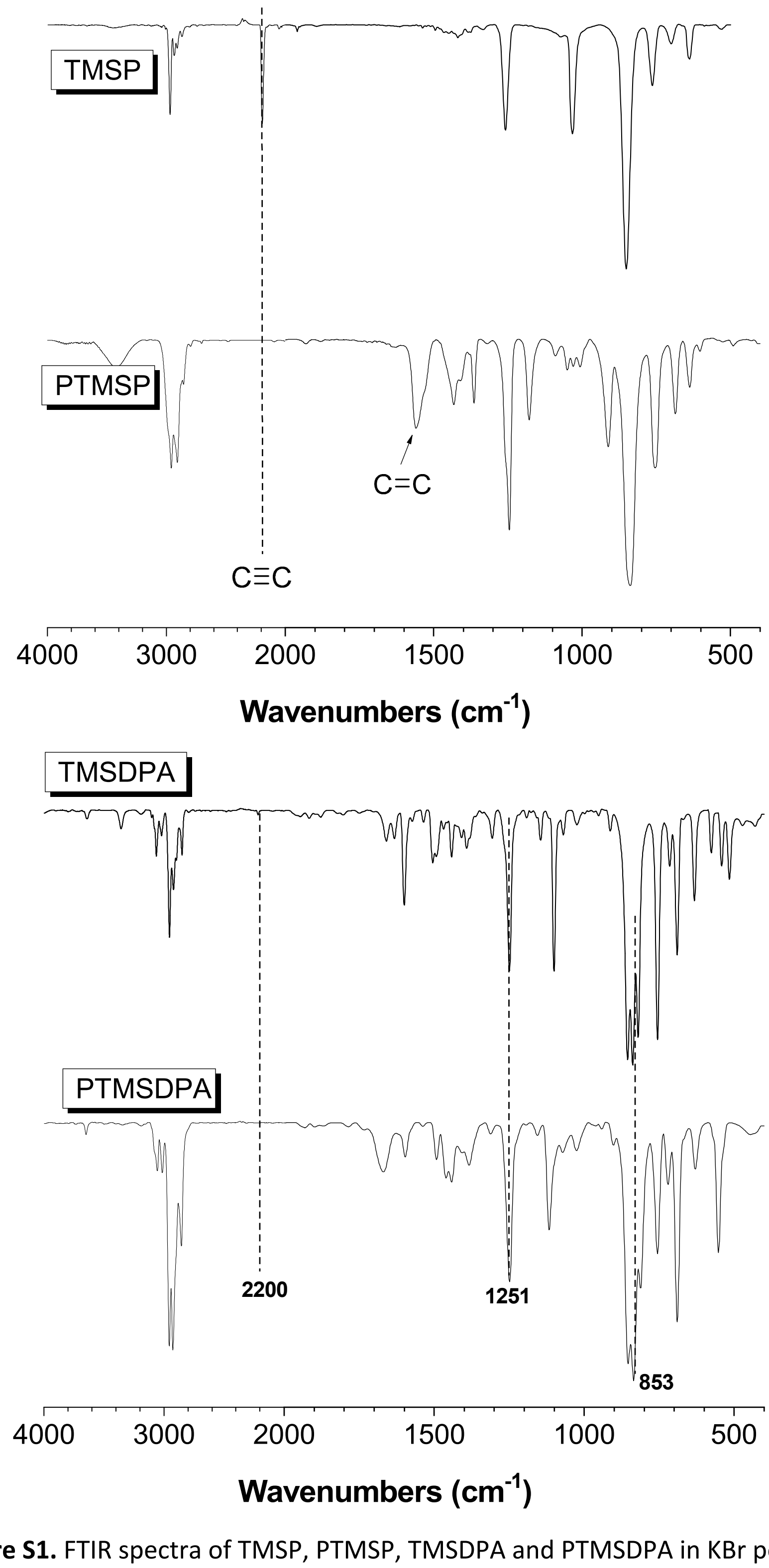

Figure S1. FTIR spectra of TMSP, PTMSP, TMSDPA and PTMSDPA in KBr pellets.

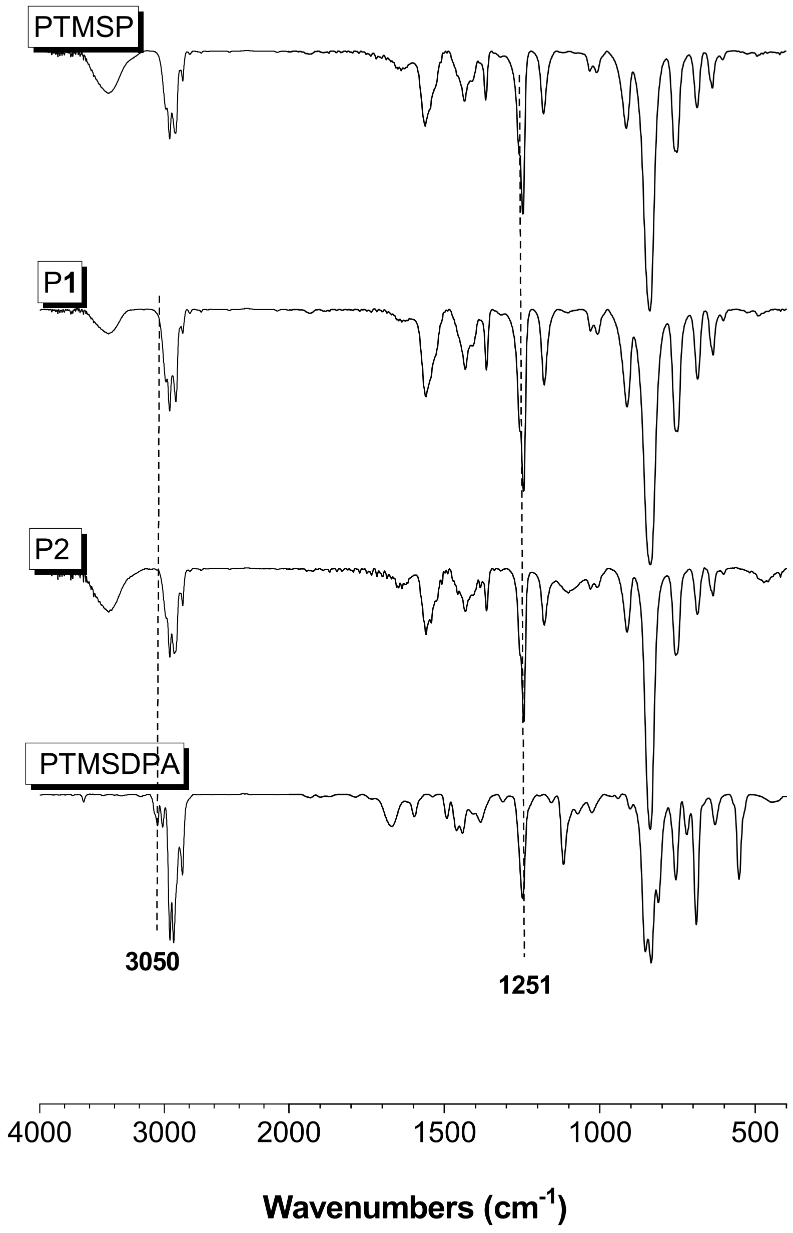

Figure S2. FTIR spectra of PTMSP, P**1**, P**2** and PTMSDPA in KBr pellets.

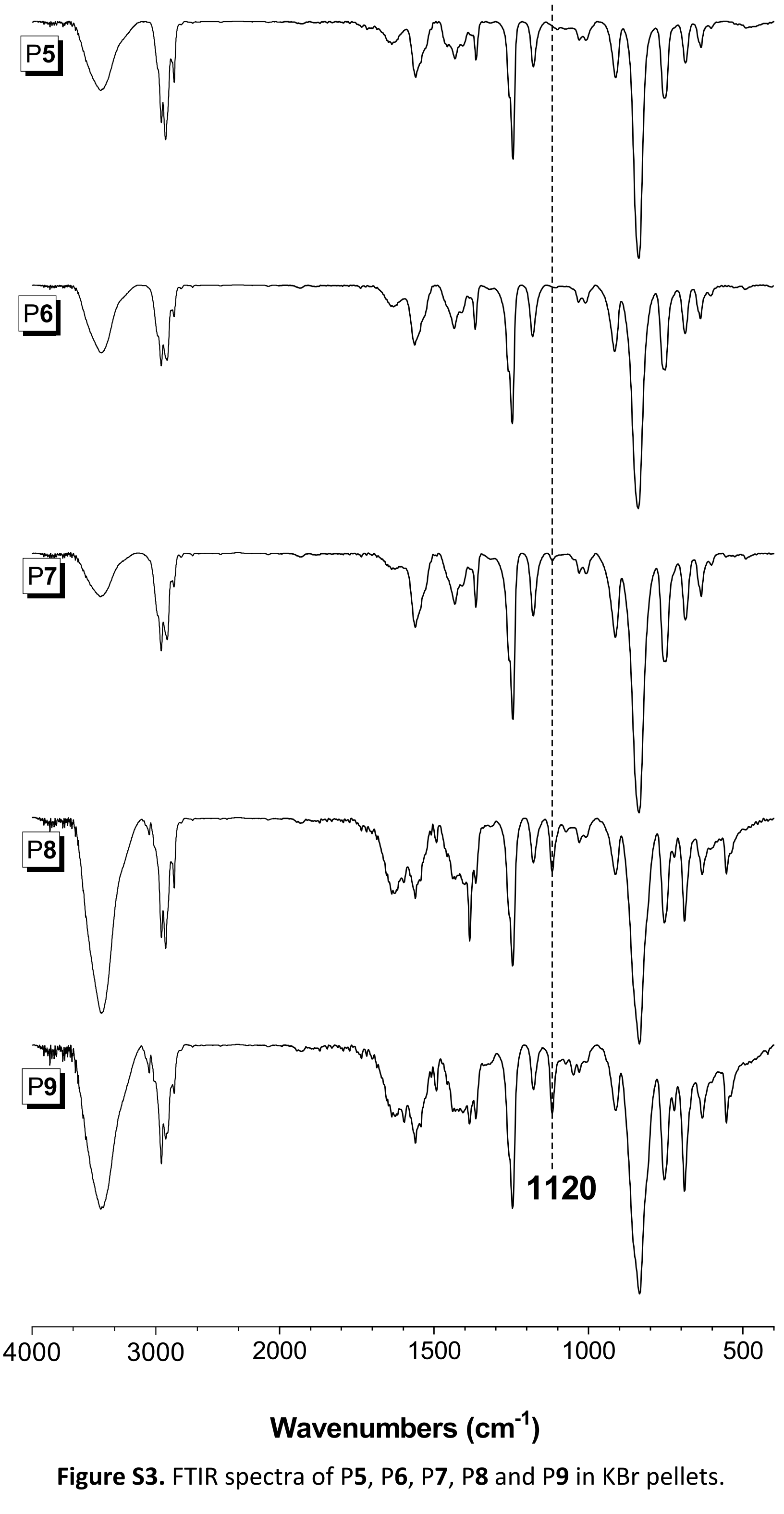

Figure S3. FTIR spectra of P**5**, P**6**, P**7**, P**8** and P**9** in KBr pellets.

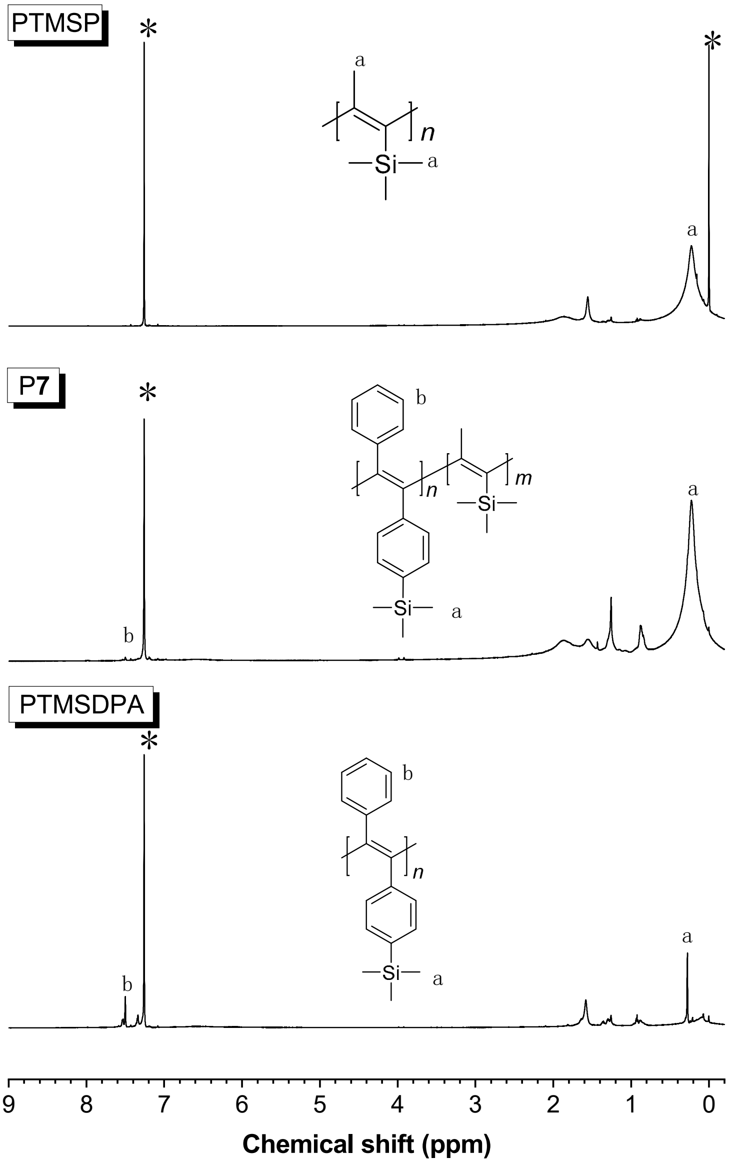

Figure S4. ^1H NMR spectra of PTMSP, P**7** and PTMSDPA ($CDCl_3$). The asterisks represent solvent peaks.

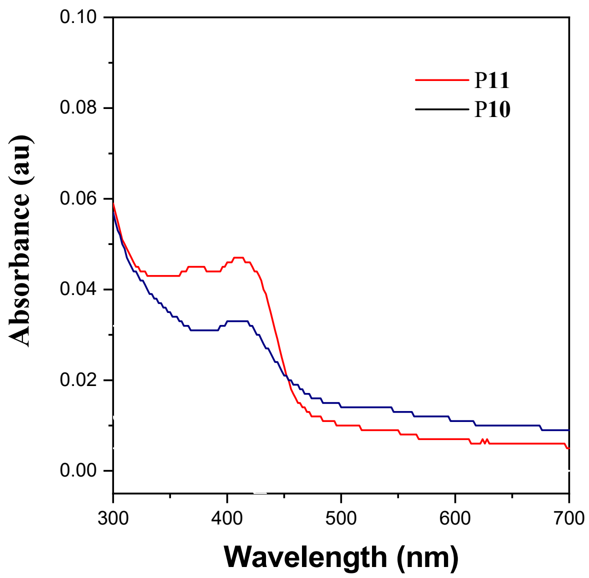

Figure S5. UV-vis spectra of **P10** and **P11** in THF solution with concentration of 10 μM.

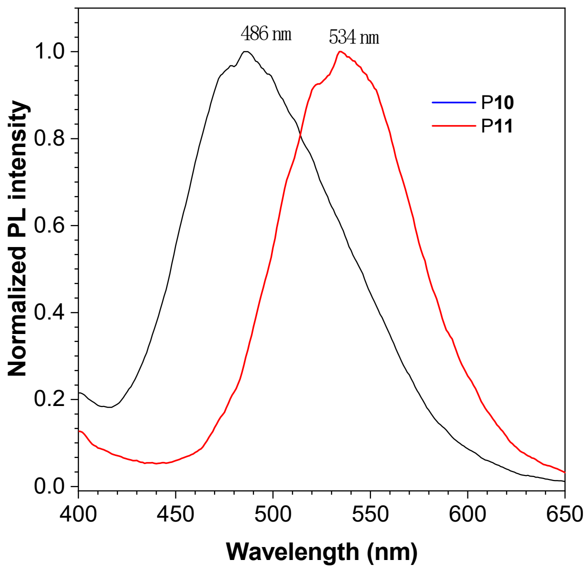

Figure S6. PL spectra of P**10** and P**11** powders. λ_{ex} = 375 nm.

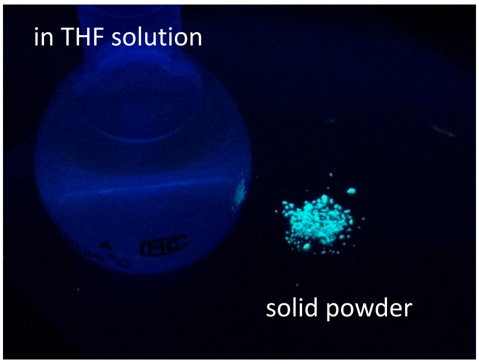

Figure S7 Fluorescence photos of P**6** in THF solution (10^{-3} M) and powder under 365nm light.

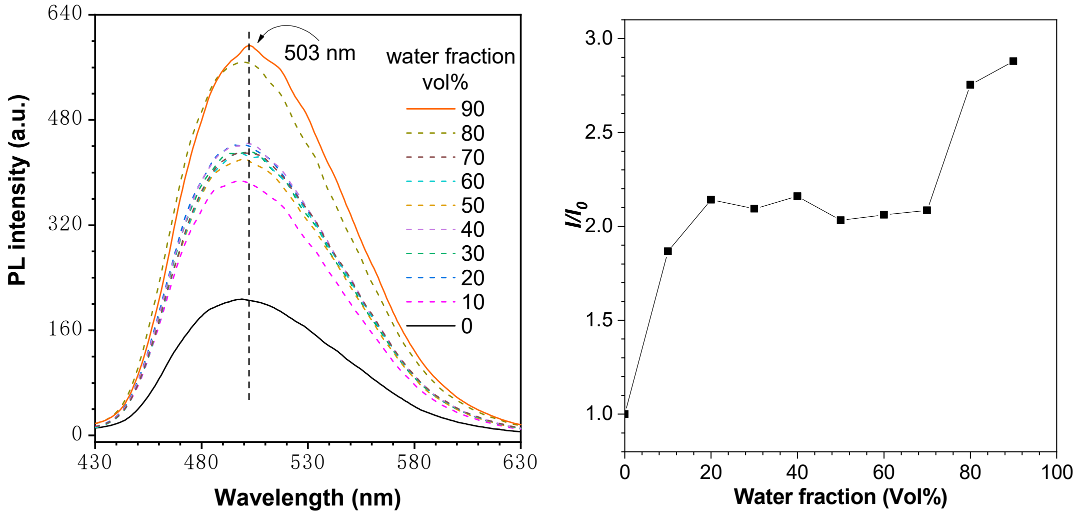

Figure S8. (Left) PL behavior of P**7** in THF/water mixtures with different water fractions (f_w). (Right) Plot of peak intensity of P**7** in THF/water mixtures with different water fractions. I_0 = Peak intensity of P**7** when f_w = 0; I = Peak intensity of P**7** when $f_w \neq 0$. λ_{ex} = 375 nm, Concentration = 100 μM.

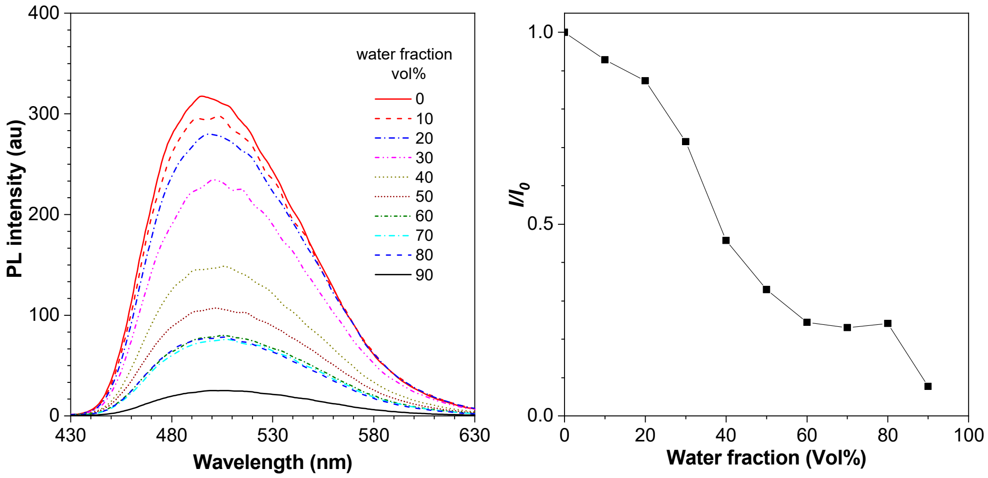

Figure S9. (Left) PL behavior of PTMSDPA in THF/water mixtures with different water fractions (f_w). (Right) Plot of peak intensity of PTMSDPA in THF/water mixtures with different water fractions. I_0 = Peak intensity of PTMSDPA when f_w = 0; I = Peak intensity of PTMSDPA when $f_w \neq 0$. λ_{ex} = 375 nm, Concentration = 100 μM.

Table S1. The quantum yield for the suspensions of PTMSDPA, P**6** and P**7** [a] .

Samples	Water fraction (%)	QY(%)
PTMSDPA	0	21.5
PTMSDPA	90	28.3
P**6**	0	-
P**6**	90	20.8
P**7**	0	24.9
P**7**	90	17.8

[a] QY = quantum yield. [M] = 100 μM. λ_{ex} = 375 nm.
